# Individual Winter Movement Strategies in Two Species of Murre (*Uria* spp.) in the Northwest Atlantic

**DOI:** 10.1371/journal.pone.0090583

**Published:** 2014-04-02

**Authors:** Laura A. McFarlane Tranquilla, William A. Montevecchi, David A. Fifield, April Hedd, Anthony J. Gaston, Gregory J. Robertson, Richard A. Phillips

**Affiliations:** 1 Cognitive and Behavioural Ecology, Department of Psychology, Memorial University of Newfoundland, St. John's, Newfoundland and Labrador, Canada; 2 Environment Canada, National Wildlife Research Centre, Carleton University, Ottawa, Ontario, Canada; 3 Wildlife Research Division, Environment Canada, Mount Pearl, Newfoundland and Labrador, Canada; 4 British Antarctic Survey, Natural Environment Research Council, Cambridge, United Kingdom; Norwegian Polar Institute, Norway

## Abstract

Individual wintering strategies and patterns of winter site fidelity in successive years are highly variable among seabird species. Yet, an understanding of consistency in timing of movements and the degree of site fidelity is essential for assessing how seabird populations might be influenced by, and respond to, changing conditions on wintering grounds. To explore annual variation in migratory movements and wintering areas, we applied bird-borne geolocators on Thick-billed Murres (*Uria lomvia*, n = 19) and Common Murres (*U. aalge*, n = 20) from 5 colonies in the Northwest Atlantic for 2–4 consecutive years. Thick-billed Murres ranged widely and among-individual wintering strategies were highly variable, whereas most Common Murres wintered relatively near their colonies, with among-individual variation represented more by the relative use of inshore vs. offshore habitat. Within individuals, some aspects of the wintering strategy were more repeatable than others: colony arrival and departure dates were more consistent by individual Common than Thick-billed Murres, while the sizes of home ranges (95% utilization distributions) and distances travelled to wintering area were more repeatable for both species. In consecutive years, individual home ranges overlapped from 0–64% (Thick-billed Murres) and 0–95% (Common Murres); and the winter centroids were just 239 km and 169 km apart (respectively). Over the 3–4 year timescale of our study, individuals employed either fixed or flexible wintering strategies; although most birds showed high winter site fidelity, some shifted core ranges after 2 or 3 years. The capacity among seabird species for a combination of fidelity and flexibility, in which individuals may choose from a range of alternative strategies, deserves further, longer term attention.

## Introduction

Many seabirds migrate seasonally to wintering areas where foraging and environmental conditions, affected by natural and anthropogenic processes, can influence their population dynamics [1], [2]. These influences can be direct, through mortality, or indirect, through carry-over effects of winter body condition on subsequent reproduction [3], [4]. Individual migratory strategies appear to vary considerably among species. For example, most birds from the same breeding population may migrate to the same region [5], [6], or portions of the population may migrate to different regions [7]–[9]. Similarly, during successive years individuals may take one or several different routes to reach these areas [10]–[14]. Variation in individual movement strategies or winter distribution can be linked to colony of origin, sex, age, experience, or breeding status [2], [9], [14], [15]. Thus, information on individual movement strategies, both within and among years, can highlight the relative consistency in use of migratory corridors and in discrete wintering grounds which may need conservation attention [1], [7]. Among the few studies of seabirds that tracked the same birds repeatedly, some species showed high wintering-site fidelity [1], [5], [12], [14], whereas in others, individuals shifted wintering locations between years [16]–[18]. Furthermore, the degree of flexibility in destination, travel times, timing of departure to and arrival at wintering sites can vary by species and is dependent on particular environmental or energetic constraints [19]. The extent of individual flexibility in wintering strategies provides valuable insight into selection pressures within and between populations [10], [16], [19], [20]. Individual behavioural flexibility will influence the capacity of populations to cope with rapid climatic and habitat changes [17], [21].

The aim of this study was to assess individual consistency in wintering strategy in two closely related species, Thick-billed Murre (*Uria lomvia*) and Common Murre (*U. aalge*). Both species are abundant in the northern hemisphere and exhibit contrasting migratory strategies: Thick-billed Murres tend to migrate longer distances from high-latitude breeding sites to lower-latitude wintering areas, whereas Common Murres make much shorter-distance migrations [22]–[25]. Even in cases where the species breed sympatrically and wintering areas overlap [23], Thick-billed Murres tend to move more, with wider distributions during spring [LMT, unpulished data]. Adult survival, breeding success, and population size have been linked to winter conditions in both species [26]–[28], underlining the intense selection pressures and consequences of behavioural decisions during the nonbreeding period. Our objective was to determine the degree of consistency in migration strategies by individuals in successive years by determining if they 1) arrive or depart colonies on similar dates, 2) travel the same distances, and 3) winter in the same areas; and 4) we investigated whether these patterns differed between species. We discuss implications of varying degrees of repeatability in particular components of the wintering strategy and their relationships with migratory connectivity.

## Materials and Methods

### Ethics statement

Fieldwork was carried out under a Government of Nunavut Wildlife Research Permit NUN-SCI-08-55, Canadian Wildlife Service Migratory Bird Banding Permit WAM-10322, and Animal Care Committee Permits 0800AG01 (Environment Canada) and WM-01-11 (Memorial University). Newfoundland and Labrador Parks Division granted access to the Provincial Seabird Ecological Reserves at the Gannet Islands, Funk Island, and Witless Bay Islands.

### Study Area

**Figure 1:**
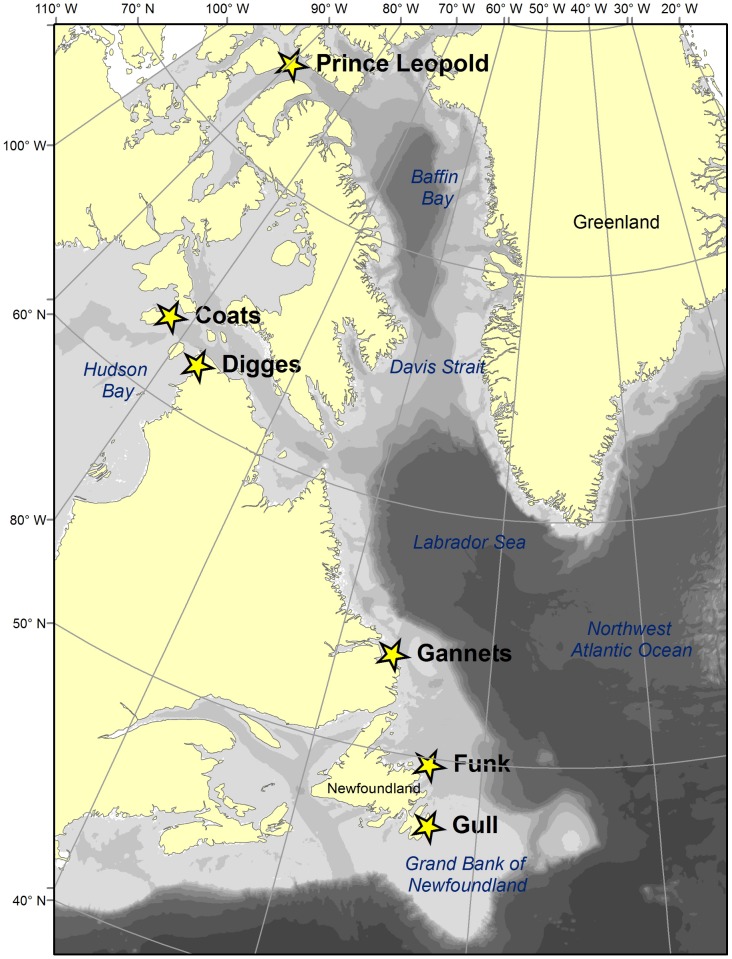
Study area and colonies. Study area indicating location of colonies of Thick-billed Murres (Prince Leopold, Coats, Digges, and Gannet islands) and Common Murres (Gannet, Funk, and Gull islands).

As part of a larger study [23], Thick-billed and Common Murres were captured at 6 breeding colonies spanning 47–74°N latitude in eastern Canada (Figure 1), during the summers of 2007–2011. These sites held either Thick-billed Murres or Common Murres, except at the Gannet Islands where the species breed sympatrically (Table 1). At each colony, birds (confirmed breeders by the presence of eggs or chicks) were captured from breeding cliffs and a geolocation-immersion logger (global location sensor (GLS); British Antarctic Survey Models Mk 5, Mk 7, Mk 13, Mk 15; ≤3.5 g) was attached to the leg using a band and cable ties (mass including the logger was ≤5.4 g, equivalent to ≤0.6% body mass). The logger was replaced in birds that were recaptured in the following year in order to track the same individual repeatedly; some elusive individuals were retrieved 2–3 years after the device was attached.

**Table 1:** Details of collection of GLS tracking devices among repeat-tracked individuals of Thick-billed and Common Murres at each study colony.

Species	Colony	Latitude, Longitude	Years Data Collected	Number of Repeat Individuals	Number of Annual Tracks
Thick-billed Murre	Prince Leopold	74°02′N, 90°00′W	2008–10	1	2
	Coats	62°53′N, 82°00′W	2007–10	8	17
	Digges	62°32′N, 77°45′W	2008–10	3	6
	Gannets	53°56′N, 56°32′W	2008–11	7	18
Common Murre	Gannets	53°56′N, 56°32′W	2008–11	7	12
	Funk	49°45′N, 53°11′W	2007–11	6	18
	Gull	47°16′N, 52°46′W	2007–11	7	14
*Overall*			*2007–2011*	*39*	87

Data were retrieved 1, 2, or 3 years following deployment.

### Data processing

GLS data were processed, filtered, and smoothed twice to determine year-round spatial distribution (refer to [23] for a full description). Filtered GLS data are archived at www.movebank.org. Year-round tracks were assessed individually to describe general winter movement. To determine the timing of the start and end of annual migration, colony arrival and departure dates were estimated using immersion (wet/dry) data. The loggers test for saltwater immersion every 3 sec and log either the total number of positive tests at 10-minute intervals, or every change of state from wet to dry and *vice* versa exceeding >6 sec). The subsequent pattern of wet/dry activity was particularly helpful in defining colony attendance, especially colony departure dates in late summer (when light data from the GLS device is affected by approach of the vernal equinox [29]), and for detailing colony attendance at high latitudes when light data is adversely affected by very long daylengths (i.e one cannot calculate the timing of sunset when there is no sunset [30]). To reduce the possibility that observed dry periods were due to extended periods of flight (i.e. during migration to or from colonies), only those lasting >6 h at the appropriate time of year were presumed to indicate birds attending colonies. In the absence of immersion data (in some cases, GLS devices recorded light but not immersion because the relevant memory sector was full, or there was partial device failure), individual locations were mapped in a GIS and colony departures and arrivals were presumed to reflect dates of final exit or initial entry of the area within a 185-km radius of the colony (similar to the mean geolocation error [31]). Due to erroneous positions generated by light shading at the colony, this approach was less precise so it was used only when wet/dry activity data was absent. Comparisons of arrival and departure dates in consecutive years were standardized according to the mean dates (± SD), for each colony in each year. We were thus discerning whether individual birds were relatively early or late. Colony departure information on the Gannet Islands was excluded for 2010, when a polar bear *Ursus maritimus* was present; but was included for Funk Island in 2009 and 2010, when an arctic fox *Vulpes lagopus* was present at Funk Island [32], as colony attendance timing there was not detectably affected (LMT unpublished data). As well, despite differences in life history, colony arrival and departure times did not differ detectably between male and female Thick-billed Murres, and differed by only 3 days in Common Murres (LMT unpubl.data). Sex was therefore not included as an explanatory variable in individual colony attendance patterns in this analysis.

### Spatial calculations

To facilitate comparisons of winter areas, data were restricted to January (mid-winter) when all birds had reached wintering grounds and GLS positions were maximally dense, indicating that birds remained resident in the same area. Centroids of the locations of each bird in January each year were calculated using ESRI ArcMap 10.1 (Spatial Statistics Toolbox). Great-circle distances, straight from the colony of origin to each winter centroid, were calculated using ArcMap. Distances along migration paths (i.e from colony to winter area) could not be accurately measured due to equinox effects on GLS loggers during fall migration period (see above); straight distances from colony to wintering area will still allow a relative comparison of the repeatability of distances to winter centroids. Wintering areas for each individual in each year were considered to be those within the 50% and 95% utilization distributions or kernel contours (KHR), calculated from the GLS point data in January and using LSCV smoothing, in the “adehabitatHR” [33] package in R (version 2.15.2 [34]). All KHRs were created using the same smoothing function. The 50% and 95% KHR are referred to below as the core and home ranges, respectively. All means are presented ± SD.

### KHR Overlap

The use of KHR highlighted areas of concentrated use for each individual in January. Birds were considered to have shifted distribution from one year to the next when core ranges (50% KHR) did not overlap.

Overlap of winter ranges (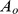) (for 50% and 95% KHRs separately) was calculated as the area of KHR in year *j* that overlapped the area in year *j*+1, and subtracted from the total area (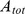):
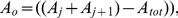
(1)recognizing that in some cases, A*_o_* = 0 (i.e. the area did not overlap).

As well, to maintain sensitivity to changes in range size between years (cf. [14]), annual Percent Habitat Overlap (P*_j_*) was also calculated for each year (e.g. year 1 on year 2, and year 2 on year 1), as:
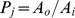
(2)


### Repeatability statistics

Following approaches by Fifield et al. [14], individual repeatability in consecutive years was calculated for five aspects of wintering strategy: 1) timing of colony departure and arrival to determine start and endpoints of migration; 2) distance travelled between colony and winter (January) centroids; 3) the size (km^2^) of individual core (50% KHR) and home ranges (95% KHR); 4) distance between annual centroids; and 5) percent overlap of kernel core and home ranges. Repeatability of these aspects was measured using three approaches: first, using linear mixed-effect models (lme; with individual and colony set as random factors, and using conditional R^2^ values [35]) to assess relationships of variables (i.e. timing, distance, overlap) between successive years. Second, an intra-class correlation coefficient (ICC) [36] was used to quantify among-groups variance (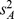) and within-individual variance 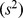 components, where repeatability (*r*) is calculated as:
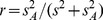
(3)High *r* scores indicate consistent behaviour, since the greatest variance occurs among, not within, individuals [36]. Variance components (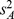 and 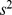), were derived from lme models for each colony separately, using R (ICC.lme in library {psychometric}). Third, inter-centroid distances and KHR overlaps were compared to randomized distributions (n = 10,000 randomizations) using Kolmogorov-Smirnov (KS) tests and also comparing the median of distributions (cf. [14]). Randomized distributions were created by drawing 10,000 random samples of two KHRs (for overlap) or two points (for centroids) using a bootstrap resampling function in R, and calculating the subsequent random overlap, or random distance (respectively). In addition, randomizations of KHR overlaps and centroids were created using a larger dataset of tracked individuals from the same colonies (n = 112; see [23]), many of which were tracked only once, but which were tracked during the same range of years as the repeat individuals (2007–2011). As well, the sample of tracked birds is representative of the spatial diversity in wintering areas as the larger sample. Because of inherent differences in movement between them, randomizations were done separately for each species, and species were considered separately for each repeatability analysis.

## Results

**Figure 2:**
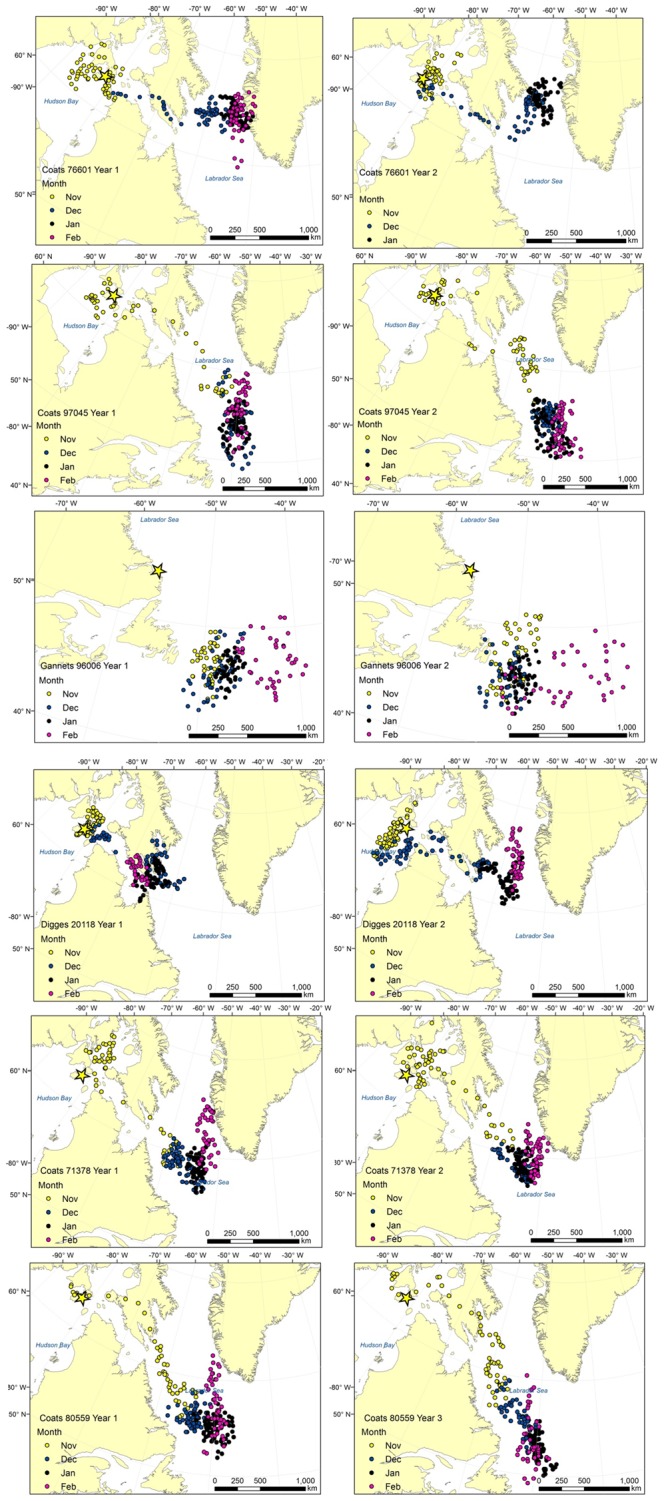
Examples of repeated positions. Examples of repeated winter positions of 6 Thick-billed Murres from Coats, Digges, and the Gannet islands. Consecutive years are presented horizontally for each individual bird, and numbers (e.g. 76601) identify individuals. Note GLS records stopped in January for Coats 76601 in year 2; and years for Coats 80559 are not consecutive, but two years apart.

Thirty-nine individuals (19 Thick-billed Murres, 20 Common Murres) were tracked for 2–4 consecutive years, providing a total of 87 annual tracks (Table 1). Detailed examples of consistent monthly movements in consecutive winters for six individual Thick-billed Murres are provided in Figure 2. Some inter-annual variation in monthly positions occurred (eg. Figure 2, Digges 20118) but overall, wintering patterns were very similar across years. Quantitative assessment of travel timing, distances, KHR sizes, and regional fidelity are presented as follows:

### Timing of migration

**Figure 3:**
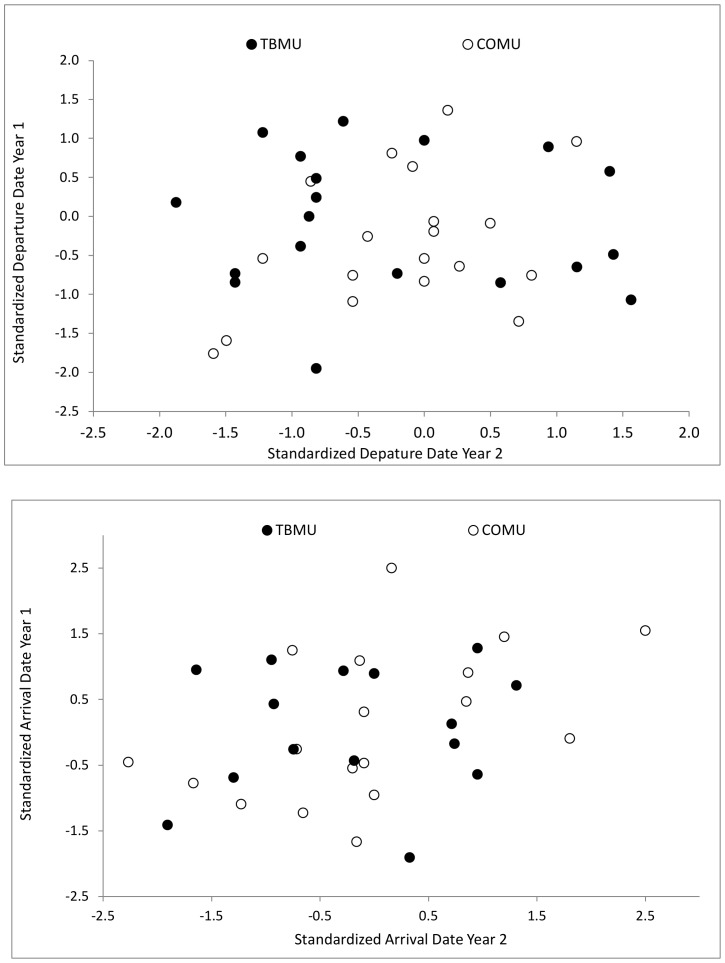
Timing of colony departures and arrivals. Consistency in timing of (A) colony departure and (B) colony arrival in consecutive years, for Thick-billed Murres (TBMU) and Common Murres (COMU). Dates are standardized to colony means, indicating whether early (or late) individuals were more likely to do the same in successive years.

Relative departure dates of individuals (standardized to annual means for each colony) were not correlated between consecutive years in either species (Thick-billed Murres, lme, F_1,5_ = 1.98, p = 0.22, R^2^ = 0.23; Common Murres, lme, F_1,11_ = 3.7, p = 0.08, R^2^ = 0.24; Figure 3a). Similarly, the repeatability (*r*) of standardized departure dates was low for Thick-billed Murres (*r* = 0) and high only for Common Murres from Gannet and Funk Islands (*r* = 0.56, 0.82, respectively; Table 2). Departure dates between consecutive years differed on average by 6.7±5.3 days (range 2–27 days) in Thick-billed Murres and by 5.7±4.6 days (range 0–16 days) in Common Murres.

**Table 2:** Repeatability (r), measured through intra-class correlation coefficients, among wintering parameters of Thick-billed and Common Murres (calculated separately for each colony).

	Repeatability (r)					
Wintering Parameter	N	Thick-billed Murre		N	Common Murre	
Colony departure date (standardized)	17	Coats	<0.01	6	Gannets	0.56
	6	Digges	<0.01	18	Funk	0.82
	10	Gannets	<0.01	10	Gull	<0.01
Colony arrival date (standardized)	12	Coats	0.18	16	Gannets	0.35
	4	Digges	0.00	16	Funk	0.42
	14	Gannets	0.04	8	Gull	0.64
Distance to winter centroid (km)	18	Coats	0.40	18	Gannets	0.32
	6	Digges	0.46	22	Funk	0.29
	20	Gannets	<0.01	14	Gull	0.61
Size of 50% KHR (km^2^)	16	Coats	0.19	20	Gannets	<0.01
	6	Digges	0.29	26	Funk	0.07
	18	Gannets	0.54	14	Gull	0.19

High scores of r indicate relatively consistent individual behaviour. N indicates number of repeat measurements.

Although standardized arrival dates in consecutive years (year one vs. year two) were not correlated in Thick-billed Murres (lme, F_1,15_ = 0.07, p = 0.93, R^2^ = 0.001), or Common Murres (lme, F_1,11_ = 4.3, p = 0.06, R^2^ = 0.25; Figure 3b), compared to departure dates, arrival dates of individuals were generally more repeatable, but still lower for Thick-billed Murres (*r* = 0–0.18) than for Common Murres (*r* = 0.34–0.64; Table 2). Arrival dates between consecutive years differed on average by 11.5±8.1 days (range 2–35 days) in individual Thick-billed Murres, and by 12.6±9.3 days (range 1–32) in individual Common Murres.

### Winter centroids

#### Travel distances

**Figure 4:**
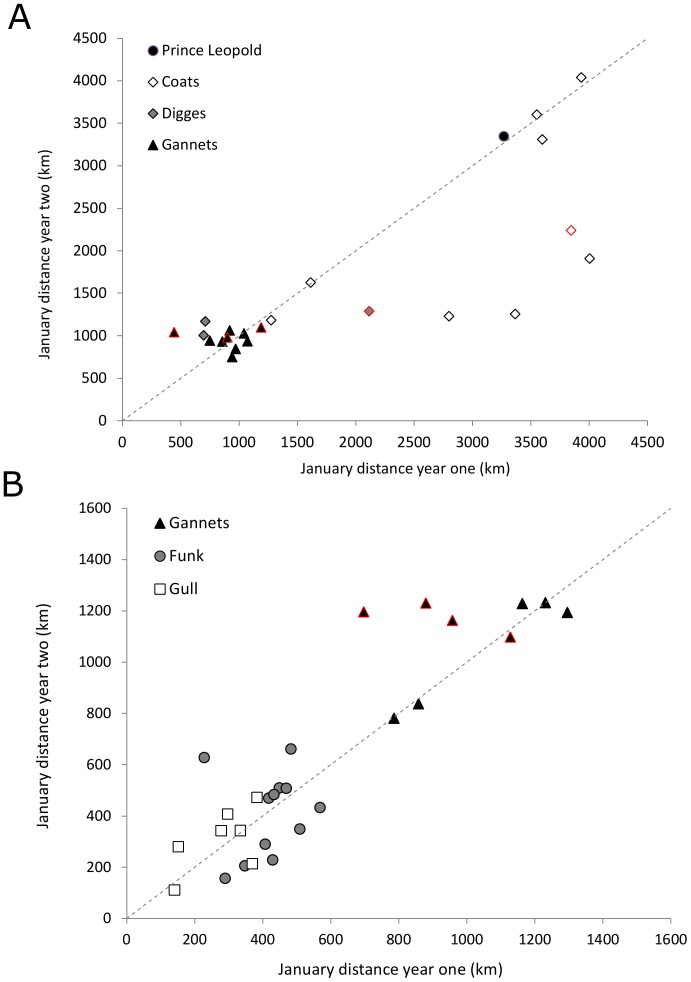
Repeated distances travelled. Relationship of distance travelled (straight line, in km) from the colony to the January centroid in consecutive years (year one vs. year two), for (A) Thick-billed Murres and (B) Common Murres. Dotted line represents the hypothetical 1∶1 relationship if distances are the same in successive years. Markers outlined in red indicate birds that switched regions across years.

Distances travelled to consecutive winter centroids were highly correlated, in both Thick-billed Murres (lme, F_1,16_ = 3.44, p<0.003, R_c_^2^ = 0.66; Figure 4a) and Common Murres (lme, F_1,24_ = 8.96, p<0.006, R_c_^2^ = 0.78; Figure 4b). Similarly, distances travelled to consecutive winter centroids were repeatable (depending on colony) for both Thick-billed Murres (*r* = 0.0–0.46) and Common Murres (*r* = 0.32–0.61; Table 2). Six Thick-billed Murres (particularly from Coats and Digges islands) travelled shorter distances in the second winter (Figure 4a); yet a change in distance travelled (Figure 4; points outlined in red) did not always indicate a shift in distribution (i.e. no 50% KHR overlap) between years.

#### Inter-centroid distance

The median distance between consecutive winter centroids was small, differing by 239 km (range 22–1212 km) for Thick-billed Murres and 169 km (range 43–631 km) for Common Murres. Furthermore, in Thick-billed Murres, the median distance between consecutive winter centroids was significantly lower for birds tracked in consecutive years (239.1 km) than the distance between random pairs of centroids (897.6 km; KS test, D = 0.66, p<0. 0001). Similarly in Common Murres, the median distance between consecutive winter centroids was significantly lower for birds tracked repeatedly (169.8 km) than randomly-paired centroids (333.2 km; KS test, D = 0.51, p<0. 0001; Figure S1, S2).

### Winter distributions

#### Range sizes

**Figure 5:**
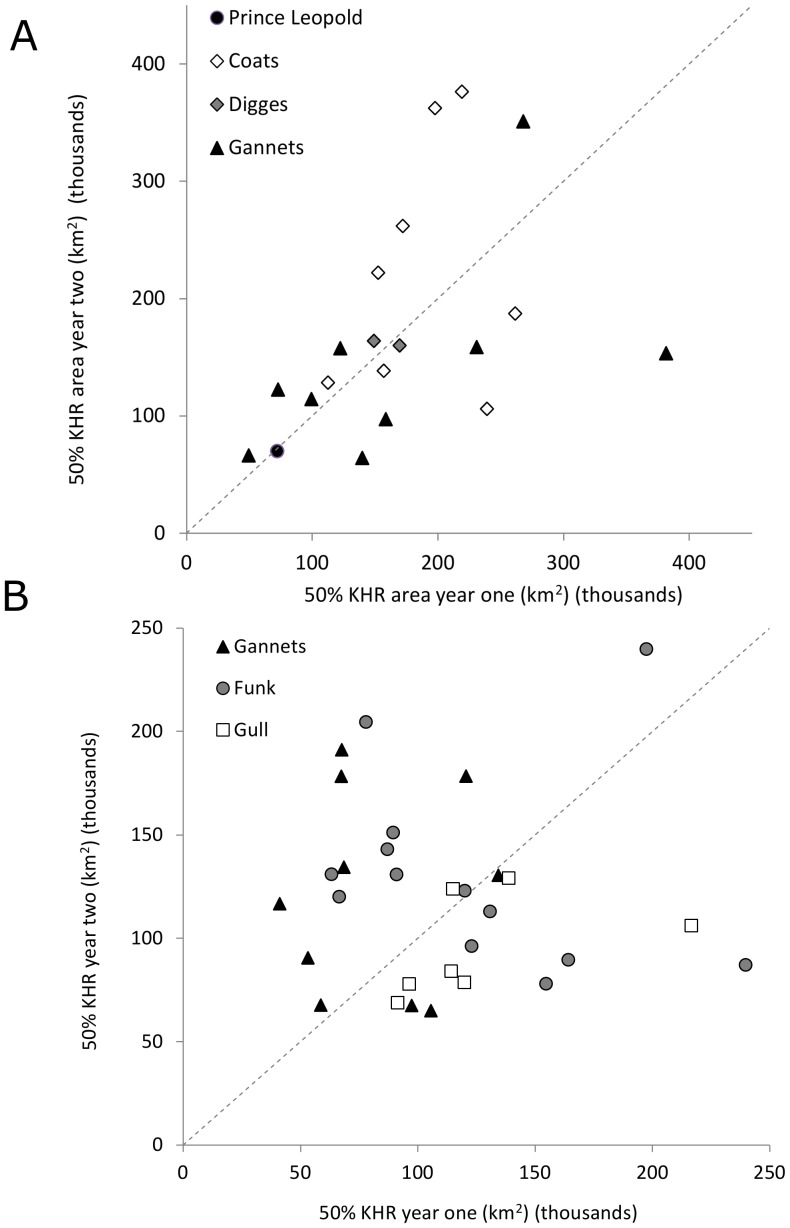
Repeated home range sizes. Relationship of 50% KHR core range sizes (km^2^, in thousands) in consecutive years (year one vs. year two), for (A) Thick-billed Murres and (B) Common Murres. Dotted line represents the hypothetical 1∶1 relationship if core sizes are the same in successive years.

The size of core ranges (50% KHR) was positively but not strongly correlated between consecutive years, in either Thick-billed Murres (lme, F_1,16_ = 3.98, p = 0.06, R^2^ = 0.17; Figure 5a) or Common Murres (lme, F_1,24_ = 0.07, p = 0.93, R^2^ = 0.02; Figure 5b), and variance appeared to be higher for individuals with larger core ranges (Figure 5). Yet repeatability (*r*) in the size of core ranges (50% KHR) was relatively higher for Thick-billed Murres (*r* = 0.19–0.54) than for Common Murres (*r* = 0–0.19; Table 2).

#### Spatial distribution

**Figure 6:**
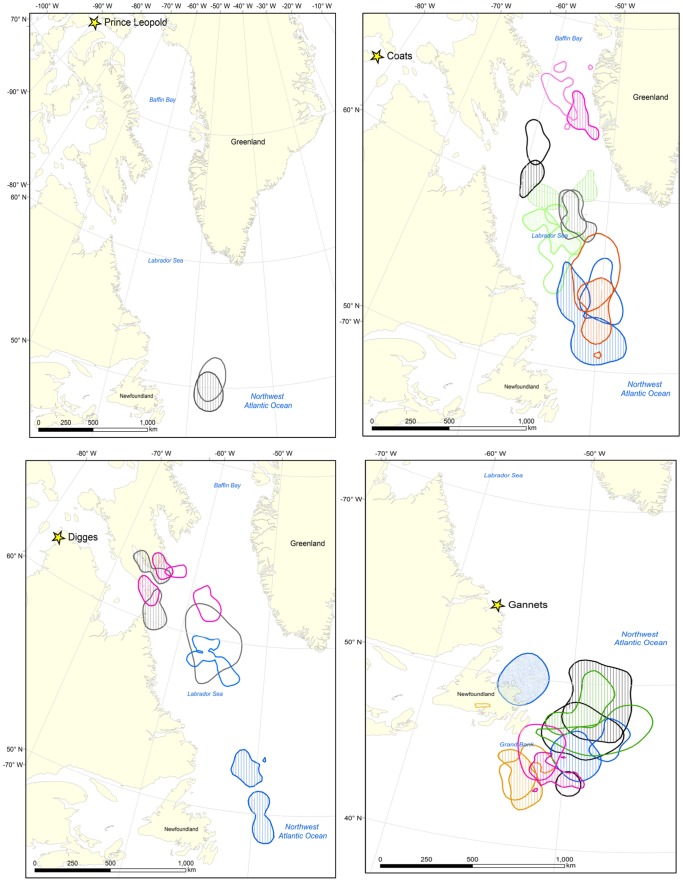
Repeated core winter areas of Thick-billed Murres. Examples of core winter areas (50% KHR) of individual Thick-billed Murres from four colonies (Prince Leopold, Coats, Digges, and Gannet islands), tracked across consecutive years. Colors note repeated observations for the same individual, with cross-hatching to identify the difference between years. To facilitate interpretation in areas of high kernel overlap, only a selection of repeat tracks is shown.

**Figure 7:**
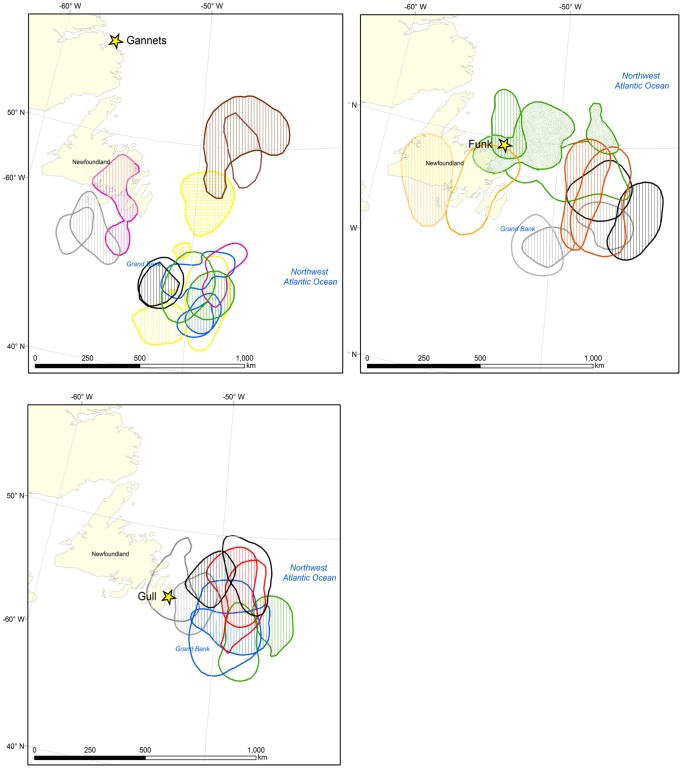
Repeated core winter areas of Common Murres. Examples of core winter areas (50% KHR) of individual Common Murres from three colonies (Gannet, Funk, and Gull islands), tracked across consecutive years. Colors note repeated observations for the same individual, with cross-hatching to identify the difference between years. To facilitate interpretation in areas of high kernel overlap, only a selection of repeat tracks is shown.

Although the majority of individuals maintained the same migration strategies (see also Figure 2), others shifted wintering locations between years (Figures 6, 7). For example, individual Thick-billed Murres from Coats Island showed consistent annual use of either the northern Labrador Sea and Davis Strait, or the mid-Labrador Sea, or the southern Labrador Sea (winters 2008, 2009, 2010); whereas individual Thick-billed Murres from Digges Island shifted core areas (winters 2009, 2010; Figure 6). The small sample size at Digges (n = 3) cannot be used to suggest a colony-specific bias in the propensity of individuals to show site fidelity, but rather to illustrate flexibility in core winter areas in some individuals. Among Common Murres, individuals generally followed consistent strategies of using either nearshore or offshore habitat in consecutive years (Figure 7). In both species, some individuals exhibited regional fidelity in the first two years but shifted distribution in the third (e.g. Gannet Islands, Figure 6, blue KHRs and Figure 7, yellow KHRs).

#### Wintering range overlaps

The extent of overlap of consecutive KHRs was extremely variable, ranging from 0–64% (home range) and 0–37% (core) in Thick-billed Murres, and 0–95% (home range) and 0–58% (core) in Common Murres. The extent of home range overlap was significantly lower in Thick-billed Murres (lme, F_1,31_ = 4.31, p = 0.05) and also varied significantly by colony (F_4,31_ = 2.79, p = 0.05; but only at Digges Island, t_1,31_ = −2.6, p = 0.02) and year (F_3,45_ = 3.5, p = 0.02). Core range overlap did not vary significantly by species (F_1,44_ = 3.06, p = 0.09), colony (F_4,30_ = 1.23, p = 0.32) or year (F_3,44_ = 2.01, p = 0.12). Extent of overlap was not related to KHR size (linear regression, F_1,35_ = 0.13, p = 0.72).

Despite the observed variability in relative overlap of KHRs between years, the median overlap of both home and core ranges for each species was significantly greater than that expected by chance, particularly for Common Murres (Table 3, Figures S3, S4). Notably, the similar overlaps of year 1 on year 2/year 2 on year 1 (Table 3) indicate no substantial change in range size across years. For Thick-billed Murres, median overlap of home ranges (95% KHR) of both years (46% year 1 on year 2; 48% year 2 on year 1) were significantly greater than expected by chance (0% for both years; KS tests, D = 0.64, p = 0.0001; Table 3, Figure S3). Median overlap of core ranges (50% KHR) in both years (16%) was also greater than expected by chance (0%; KS tests, D = 0.47, p = 0.001 for both years; Table 3).

**Table 3:** Average and median percent overlap of consecutive home and core ranges in January (95% and 50% KHR) of repeat-tracked Thick-billed and Common Murres, compared to a randomized distribution of overlap between individuals.

		% Overlap ± SD (median)			
Species	Group	95% KHR year 1 on 2	95% KHR year 2 on 1	50% KHR year 1 on 2	50% KHR year 2 on 1
Thick-billed Murre	Within-individual	50±28 (47)	49±32 (48)	18±21 (16)	18±18 (16)
	Randomized	17±31 (0)	na	16±31 (0)	na
Common Murre	Within-individual	68±28 (68)	67±27 (68)	36+31 (29)	37+42 (27)
	Randomized	32±29 (28)	na	33±30 (29)	na

Similar overlap of year 1 on year 2/year 2 on year 1 indicate no change in range size across years. Similar overlap of 50% and 95% randomized overlap indicate convergence of histograms to 0 (i.e. many random pairs do not overlap at all), regardless of KHR size.

Similarly, for Common Murres, consecutive home ranges overlapped more (median 68% for both years; Table 3, Figure S4), compared to randomized home range overlap (median 28%; KS test, D = 0.52, p<0.0001 for both years). Overlap of consecutive core ranges (median 29% year 1 on year 2; 27% year 2 on year 1; Table 3), was not greater than expected by chance (median 29%; KS tests, D = 0.11, p = 0.96 in both cases; Table 3).

## Discussion

Individual Thick-billed and Common Murres exhibited a relatively high degree of consistency in wintering strategies between years. Many individual murres travelled similar distances from colonies, repeatedly visited particular areas, and had similarly-sized core ranges in consecutive winters (less so for Common Murres). Yet variability, both among and within individuals, and also among some aspects of the wintering strategy (i.e. colony arrivals, departures, distances, destinations) [19], may illustrate some capacity for behavioural flexibility in both species.

### Colony attendance

The timing of departure and arrival at the colony were remarkably consistent for some individuals (arriving and departing on the same day each year), but not for others (up to ∼30 days difference between years). As a result, the relative departure dates for individuals in successive years were not correlated overall. Relative arrival dates at the colony each year were more repeatable for Common than Thick-billed Murres. In other species, timing of colony departures can be related to breeding success [5], [14], [15] and timing of arrivals can be influenced by energetic investments that carry over from the previous breeding attempt [38], thus the extent of repeatability in consecutive years could depend partly on individual breeding outcome. As well, given that timing of arrival and breeding is strongly linked to environmental conditions in many seabird species [37], [39], [40], variable local environmental conditions, particularly spring ice conditions near Thick-billed Murre colonies, likely account for low repeatability across years [14], [41]. Although all tracked murres were confirmed breeders at the time of device deployment, final breeding outcomes were not confirmed, and could not be used to account for individual consistency in arrivals or departures. As well, since colony attendance was mostly determined using GLS immersion data (dependent on having a dry logger for >6 hours; see methods), birds staging at sea (wet logger) near the colony would not be noted as attending. Any variability in the duration of staging at sea, either pre- or post-breeding, could influence the observed repeatability of attendance dates.

Studies on other long-distance migrants (Black-Browed Albatrosses *Thalassarche melanophris* [2], Northern Gannets *Sula bassana* [14], Bar-tailed Godwits *Limosa lapponica baueri* [19]) also suggest that timing is not necessarily repeatable for all components of the migration cycle (last visit to land, out-migration, periods at stopover and wintering sites, return migration etc.). That is, repeatability in timing is important for some events but not others, and is likely determined by a combination of genetic and environmental influences [14], [41]. These could include environmental cues, annual conditions at staging or stopover sites, and physiological constraints, particular to the ecology of each species. For example, ongoing consideration of movement strategies revealed colony differences in spring stopover sites among Thick-billed Murres (LMT, unpubl.data). Further study will provide insight into the degree of individual flexibility at different stages [19] in the migration cycle of murres.

### Wintering site fidelity

Compared to other measures of repeatability, distance travelled to winter sites was less variable within individuals (i.e. higher repeatability (*r*) values denote a decrease in within-individual variance, *s^2^*, compared to among-individual variance, *s^2^_A_*). This is unsurprising given that many individuals showed both site fidelity to particular wintering areas (Figures 2, 5, 6) and remarkable similarity in the centroids of distribution in January (which in many cases were closer than the average GLS error of ∼185 km [31]). Notable examples are the Thick-billed Murre from Prince Leopold Island which twice travelled ∼3200 km to a wintering site in the southern Labrador Sea (Figures 4, 6); two Thick-billed Murres from Coats Island which left Hudson Bay on the same date each year to travel to the northern Labrador Sea and Davis Strait region in two years, or to travel to the mid- and southern Labrador Sea in two years (Figures 4, 6); and Common Murres from the Gannet Islands travelling to the southeast Grand Banks in two years (Figures 2,6).

Regional site fidelity is common among seabirds, particularly long-distance migrants such as Gray-headed Albatrosses *Thalassarche chrystostoma* [1], Black-browed Albatrosses [5], Northern Gannets [14], and South Polar Skuas *Stercorarius maccormicki* [8]. We recorded a diversity of individual wintering strategies among and within species (particularly Thick-billed Murres), but varying degrees of site fidelity, with most individuals repeating and some switching winter sites between years. Dias et al. [17] demonstrated that even given remarkable flexibility in wintering sites between years, individual Cory's Shearwaters (*Calonectris diomedea*) chose the same areas more often than expected by chance. To date, few studies of nonbreeding site fidelity extend past two years, limiting the potential interpretation of repeatability. Catry et al. [42] noted a decrease in repeatability of laying date after 5 years in Great Skuas (*Stercorarius skua*), hypothesizing low repeatability (and high plasticity) of many traits in seabirds, in response to the dynamic nature of the marine environment. A time series of 5–8 years of stable isotope data from fur seal whiskers (*Arctocephalus gazelle*, *A. tropicalis*) suggested a high degree of individual consistency in the use of particular water masses across years [43]. It seems that the degree of repeatability may vary depending on the trait [19]. The capacity among seabird species for a combination of fidelity and flexibility, in which individuals may choose from a range of alternative strategies [12], [17], deserves further, longer term attention. As well, knowing both the variety of alternative strategies used by individuals, and the propensity of individuals to repeat particular strategies, will provide insight into the long-term persistence of important wintering areas for particular colonies.

### KHR size and overlap

Overlap of individual KHRs was much higher than expected by chance (Figure S3, Figure S4), yet similar to the distance travelled, the degree of range overlap (95% KHR) between years was extremely variable among individuals, ranging from 0–64% in Thick-billed Murres, and from 0–95% in Common Murres. Whereas the extent of home range (95%) overlap varied by colony and year, the extent of core overlap of individuals did not. This suggests that regional or environmental effects that vary across years can influence overall home range positioning but does not significantly influence core habitat locations for most individuals.

Interestingly, the overlap of consecutive individual Common Murre core winter ranges (50% KHR) did not differ from overlap expected by chance. This may be due partly to the limited geographical extent of suitable habitat in particular areas, such as on the Grand Bank (Figures 6, 7). Between years, although overall winter ranges remain the same, the time when birds occupy particular portions of their winter range may vary (e.g. inshore in December in one year, inshore in January in another). Thus, slightly different degrees of overlap may have been observed if other periods were chosen, due to variation in temporal patterns of winter movement.

### Implications for fitness

Phenotypic plasticity, in which a diverse range of behaviours or strategies are employed by different individuals in a population, is expected to improve the capacity of the populations to adapt to environmental changes [44], [45]. In the current study, variation in winter movement patterns stemmed more from between-individual variation than from annual changes within individuals. This was particularly true among Thick-billed Murres, which showed great among-individual variation in spatial use of winter habitat (Table 2, Figure 6); among-individual variation in winter ranges among Common Murres was less striking (Table 2, Figure 7). Individual Common Murres tended to aggregate on the Grand Banks, where among-individual variation in movement may be partly bounded by marine isotherms as described by Tuck [46]). Previous research has indicated that Thick-billed Murres are dietary generalists, yet as in other cases, this generalist population is composed of both specialists (each of which adopt a subset of available strategies) and generalists (each of which employ a larger range of available strategies) [47], [48], [49]. We suggest that Thick-billed Murres may also be generalists in terms of wintering strategy, with a range of habitats selected by wintering specialists and wintering generalists. In comparison, Common Murres exhibit a more restricted (specialist) wintering strategy, and this phenotypic expression may vary little among Common Murre individuals.

In concert with increased phenotypic plasticity, Thick-billed Murres display weaker migratory connectivity; that is, the extent to which individuals of a population summer and winter in the same places [50]. This diversity of individual wintering areas has implications for demographic independence among their respective populations, i.e. anthropogenic and other environmental factors influencing birds at particular wintering sites will not affect all Thick-billed Murre populations equally [23]. In contrast, Common Murres, with individuals from all three colonies wintering in a similar location, show much lower plasticity, stronger migratory consistency (both within and between colonies), and will be more susceptible to localized disturbances in winter. Multiple-colony tracking of seabird populations will continue to be critical in identifying and managing population-level threats on wintering grounds [2], [44], [51], [52].

Behavioural plasticity that enables individuals to shift strategies or explore multiple locations in response to variable environmental conditions likely improves individual fitness [16]–[18], [44], particularly by developing spatial memory [16], [53]. Young birds that disperse further will have knowledge of more alternative wintering sites [17], [54]. While many individual murres exhibited consistent wintering strategies, some showed flexible use of different areas between years. Like other cognitively complex species, we suggest that these long-lived birds (potentially reaching 25+ years of age [55], [56]) may use their spatial memory [57] garnered from years of experience, to inform and adjust annual movement tactics [17]. Guilford et al. [12] proposed an “exploration-refinement hypothesis” for Atlantic Puffins *Fratercula arctica*, suggesting that their migration strategy develops through exploratory movements and individual learning. Additional capacity for adjusting annual movements in response to resource availability [53] or variable environmental conditions [16], [19], [45] requires further investigation. How behavioural expression is influenced by environmental constraints [19], as well as by population density and competition (cf. [48], [49]), will further help discern inter-specific differences in wintering strategy. Clearly, the advantages of plasticity strongly depend on the reliability of cues that seabirds use to make decisions in a stochastic ocean environment.

## Supporting Information

Figure S1 **Frequency distribution of (A) observed and (B) randomized distances between consecutive winter centroids for Thick-billed Murres.**
(TIF)

Figure S2 **Frequency distribution of (A) observed and (B) randomized distances between consecutive winter centroids for Common Murres.**
(TIF)

Figure S3 **Frequency distribution of of (A) observed and (B) randomized overlap of 95% KHR (range) for Thick-billed Murres.**
(TIF)

Figure S4 **Frequency distribution of of (A) observed and (B) randomized overlap of 95% KHR (range) for Common Murres.**
(TIF)
