# Supplementary figures and images for: Individual Winter Movement Strategies in Two Species of Murre (Uria spp.) in the Northwest Atlantic
Source: PLoS One. 2014 Apr 2;9(4):e90583. doi: 10.1371/journal.pone.0090583 (PMC3973664; doi:10.1371/journal.pone.0090583)

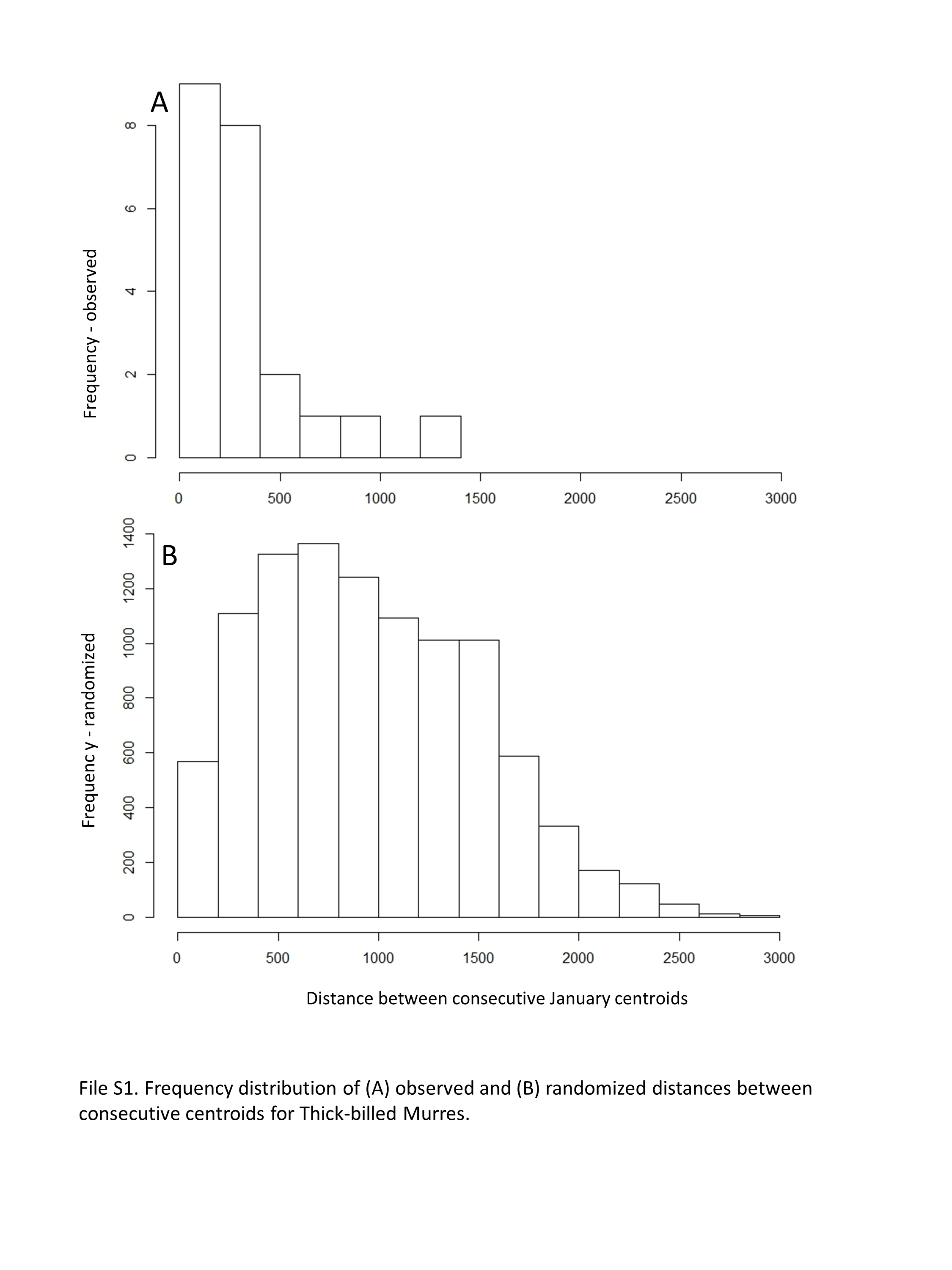

Supplement: Figure S1 — Frequency distribution of (A) observed and (B) randomized distances between consecutive winter centroids for Thick-billed Murres. (TIF) [file pone.0090583.s001.tif]

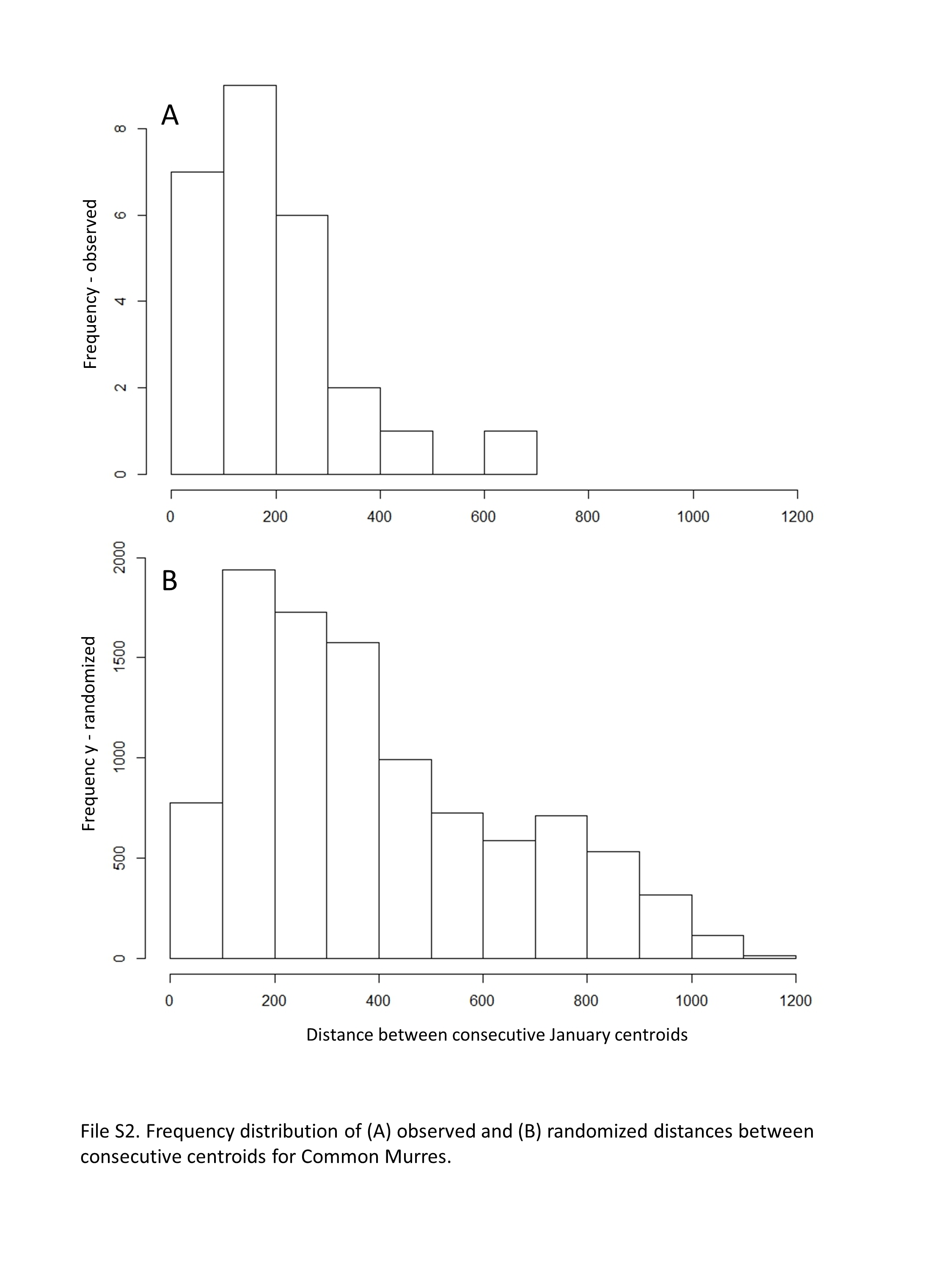

Supplement: Figure S2 — Frequency distribution of (A) observed and (B) randomized distances between consecutive winter centroids for Common Murres. (TIF) [file pone.0090583.s002.tif]

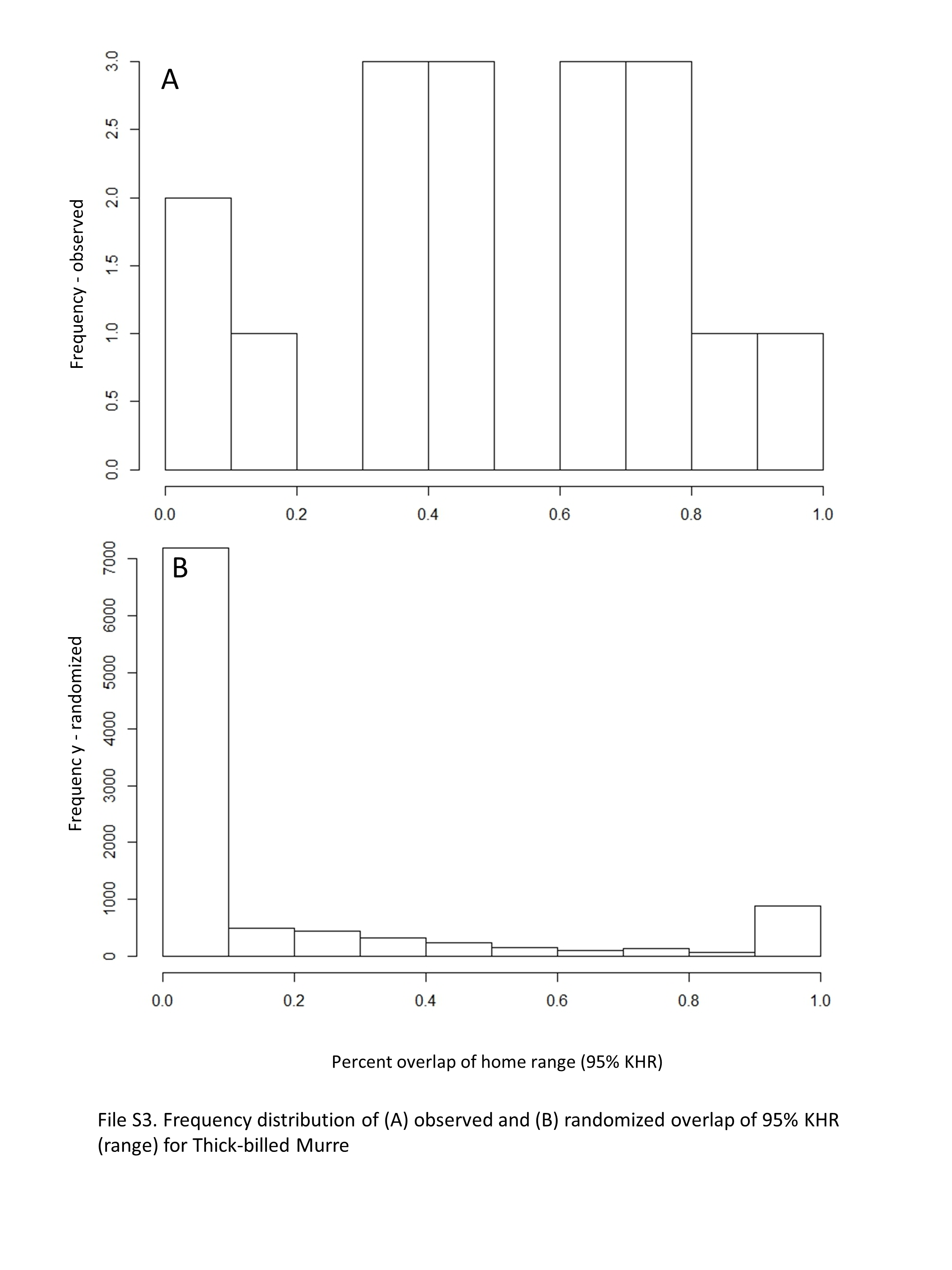

Supplement: Figure S3 — Frequency distribution of of (A) observed and (B) randomized overlap of 95% KHR (range) for Thick-billed Murres. (TIF) [file pone.0090583.s003.tif]

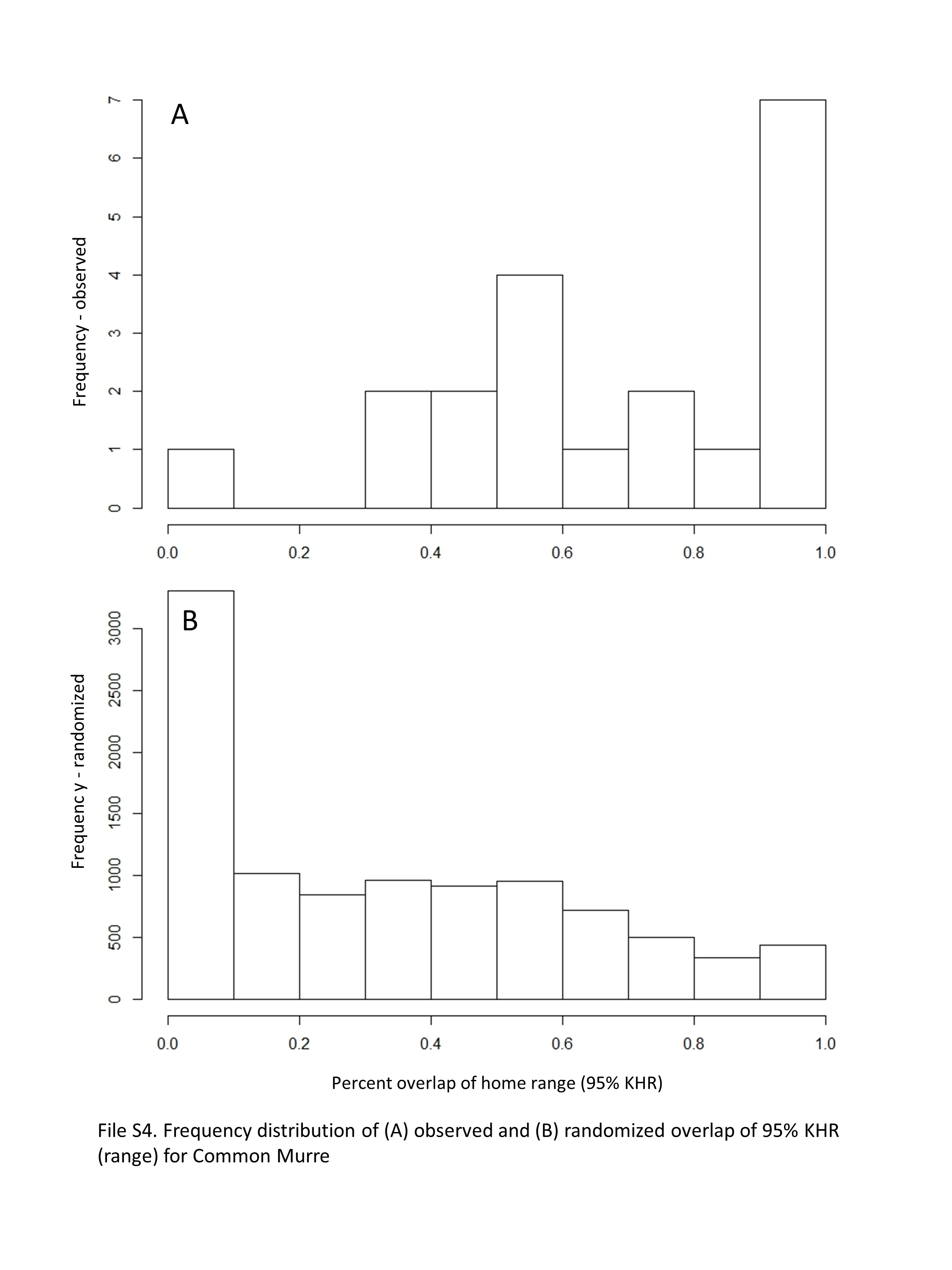

Supplement: Figure S4 — Frequency distribution of of (A) observed and (B) randomized overlap of 95% KHR (range) for Common Murres. (TIF) [file pone.0090583.s004.tif]
